# Efficacy of an inactivated bivalent vaccine against the prevalent strains of Newcastle disease and H9N2 avian influenza

**DOI:** 10.1186/s12985-017-0723-7

**Published:** 2017-03-16

**Authors:** Jing Zhao, Huiming Yang, Hongjun Xu, Zengbin Ma, Guozhong Zhang

**Affiliations:** 1 Key Laboratory of Animal Epidemiology and Zoonoses;, Ministry of Agriculture, College of Veterinary Medicine, China Agricultural University, No.2 Yuanmingyuan West Road, Haidian District, Beijing, 100193 People’s Republic of China; 2 Chengdu Tecbond Biological Products Co., Ltd, Sichuan, 610100 People’s Republic of China

**Keywords:** Newcastle disease virus, H9N2 avian influenza virus, Inactivated vaccine, Efficacy, Chicken

## Abstract

**Background:**

Newcastle disease (ND) and avian influenza subtype H9N2 (H9N2 AI) are two of the most important diseases of poultry, causing severe economic losses in the global poultry industry. Vaccination is an effective way to prevent and control the spread of ND virus (NDV) and H9N2 AI virus (AIV), but the antigenic differences between the current circulating strains and the vaccine strains might account for recent ND and H9N2 AI outbreaks in vaccinated poultry flocks.

**Methods:**

We developed an inactivated bivalent H9N2 and NDV vaccine based on the current prevalent strains of H9N2 AIV and NDV in China and evaluated its efficacy in chickens in this study.

**Results:**

The results indicated that the inactivated bivalent vaccine could induce a fast antibody response in vaccinated chickens. The hemagglutination inhibition (HI) titer in the sera increased rapidly, and the highest HI titer was observed at 4 weeks post-vaccination (wpv) with a mean titre of 8.6 log_2_ for NDV and 9.5 log_2_ for H9N2. Up until 15 wpv, HI titers were still detectable at a high level of over 6 log_2_. The immunized chickens showed no signs of disease after challenge at 3 wpv with the prevalent strains of NDV and H9N2 AIV isolated in 2012–2014. Moreover, viral shedding was completely inhibited in vaccinated chickens after challenge with H9N2 AIV and inhibited by at least 90% with NDV compared to the controls at 5dpc.

**Conclusions:**

Our findings suggest that the inactivated NDV and H9N2 vaccine induces a fast and strong antibody response in vaccinated chickens and is efficacious in poultry against NDVs and H9N2 AIVs.

## Background

Newcastle disease virus (NDV) and avian influenza virus (AIV) are two of the most important pathogens in poultry worldwide [1]. Newcastle disease (ND) is usually caused by virulent NDV, which can result in 100% mortality in many species of birds [2]. The H9N2 low pathogenic avian influenza viruses have been circulating worldwide in multiple avian species, resulting in great economic losses owing to reduced egg production or increased mortality associated with coinfection with other pathogens [3–6]. Thus, severe economic losses in the poultry industry caused by NDV and AIV highlight the importance of vaccine improvement and development.

In China, the implementation of extensive vaccination programs in commercial poultry farms has reduced the number of epizootic outbreaks of ND in recent years. However, genotype VII NDV strains of class II have remained a constant threat to domestic poultry since the 2000s and most recent Chinese isolates belong to a sublineage of genotype VIId [7–9]. The predominance of genotype VIId in China is similarly observed in other countries in Asia and the Middle East, including Iran [10], Qatar [11], Japan [12], Korea [13], Kazakhstan [14], Vietnam [15], Pakistan [16], Israel [17], and Malaysia [18, 19]. In a previous study, we genotypically characterized the NDV isolates recovered from chickens between 2005 and 2012 to establish the nature of the circulating genotype VII strains in China [20]. Our results indicated that genotype VIId strains were at least 95.1% identical at the nucleotide level across the whole genome. However, they showed only 82–83% nucleotide sequence identity with vaccine strains LaSota and B1. These vaccine strains belong to genotype II in class II and were isolated 60 years ago. The outbreaks of ND in vaccinated poultry flocks in recent years are mainly attributed to the significant differences in the biology, serology, and genetics of the predominant circulating NDV strains compared with the current vaccine strains [8].

On the basis of clade classification, 69 genotypes were detected among H9N2 chicken viruses isolated in China from 1994 to 2013 [21]. In contrast, most of the 2010–2014 viruses belonged to a single G57 genotype, which was first detected in chickens in Jiangxi and Jiangsu Provinces in 2007, and its prevalence increased sharply during 2009. Since 2010, it has been the predominant genotype in circulation throughout China. This genotype was generated by continued reassortment that can be traced back to the year 1994. The neuraminidase gene was derived from the A/chicken/Beijing/1/1994-like lineage. Since the late 1990s, vaccination has been carried out in China to prevent and control H9N2 AIV infection in chicken flocks [21]. However, commercial vaccines did not provide complete protection against endemic H9N2 AIVs [22].

In this study, we developed an inactivated H9N2 and NDV vaccine based on a H9N2 A/chicken/Hebei/G/2012 (G) virus and a ND Chicken/Shandong/aSG10/2010 (aSG10) attenuated virus that are closely related to the current endemic strains of H9N2 and ND virus in China and evaluated its efficacy in chickens.

## Results

### Challenge virus

**Fig. 1:**
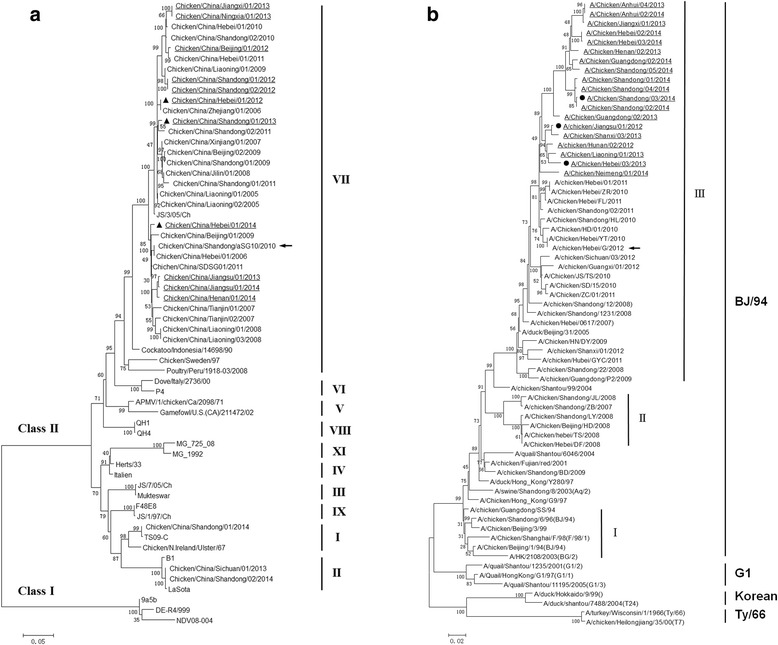
Phylogenetic tree of F genes of NDV (**a**) and HA genes of H9N2 influenza viruses (**b**). Unrooted phylogenetic trees were generated using the distance-based neighbor-joining method and MEGA 5.1 software. Statistical support for tree branches was assessed by bootstrap analysis using 1000 replications; numbers above branches indicate neighbor-joining bootstrap values that were ≥ 70%; the tree is drawn to scale, with branch lengths measured in the number of substitutions per site. Analysis was based on nucleotides of the F genes of NDV and HA genes of H9N2. The arrow indicates vaccine candidates, black triangles indicate the NDV viruses used for challenge, black circles indicate the H9 viruses used for challenge

Based on phylogenetic analysis of the NDV F gene, three NDV isolates were selected as challenge strains in this study: Chicken/China/Hebei/01/2012 (HB12), Chicken/China/Shandong/01/2013 (SD13) and Chicken/China/Hebei/01/2014 (HB14) (Table 1 and Fig. 1a). All three viruses were characterized as virulent strains based on the multiple basic amino acid motif of the fusion (F) cleavage site ^112^RRQKR↓F^117^ and classified as genotype VII NDV, which showed high genetic variation with the genotype II LaSota vaccine.

**Table 1:** Characterization of NDV and H9 AIV strains

Group	Strains	Abbreviation	Country (province)	Host	ICPI^a^	IVPI^b^	Genotype	EID_50_/0.1 mL
NDV	Chicken/Shandong/aSG10/2010	aSG10	China (Shandong)	Layer	0.25	ND	VII	10^9.30^
	Chicken/China/Hebei/01/2012	HB12	China (Hebei)	Broiler	1.96	ND	VII	10^8.50^
	Chicken/China/Shandong/01/2013	SD13	China (Shandong)	Broiler	1.89	ND	VII	10^9.30^
	Chicken/China/Hebei/01/2014	HB14	China (Hebei)	Layer	1.98	ND	VII	10^9.30^
AIV	A/chicken/Hebei/G/2012	G	China (Hebei)	Broiler	ND	0.50	BJ/94 (III)	10^9.00^
	A/Chicken/Jiangsu/03/2012	JS12	China (Jiangsu)	Broiler	ND	0.30	BJ/94 (III)	10^8.00^
	A/Chicken/Hebei/03/2013	HB13	China (Hebei)	Layer	ND	0.35	BJ/94 (III)	10^8.00^
	A/Chicken/Shandong/03/2014	SD14	China (Shandong)	Layer	ND	0.50	BJ/94 (III)	10^7.50^

*ND* no data

^a^Intracerebral pathogenicity index

^b^Intravenous pathogenicity index

Based on phylogenetic analysis of the H9N2 AIV hemagglutinin (HA) gene, three H9N2 isolates were selected as challenge strains in the study: A/Chicken/Jiangsu/03/2012 (JS12), A/Chicken/Hebei/03/2013 (HB13), and A/Chicken/Shandong/03/2014 (SD14) (Table 1 and Fig. 1b). All three viruses were characterized as LPAI H9N2 strains, which had a PSRSSR↓GLF motif in the HA gene cleavage site. Even though these three viruses and the traditional Chinese vaccine strains (A/chicken/Guangdong/SS/94, A/chicken/Shandong/6/96 and A/chicken/Shanghai/F/98) belonged to the same lineage BJ/94, there was a high degree of genetic variation among them. Lineage BJ/94 was further divided into three subgroups: I, II, and III. Of 19 H9N2 AIVs isolated between 2012 and 2014, all belonged to subgroup III and the Chinese vaccine strains belonged to group I.

### Biological characterization of the viruses

The pathogenicity of vaccine candidates and challenged strains was evaluated using the intracerebral pathogenicity index (ICPI) test in 1-day-old specific-pathogen-free (SPF) chickens and the intravenous pathogenicity index (IVPI) test in 6-week-old SPF chickens, the results are shown in Table 1. The ICPI value of the NDV vaccine strain aSG10 was 0.25, categorizing it as an avirulent (lentogenic) strain, whereas the ICPI values of the NDV challenge strains were 1.89-1.98, categorizing them as highly virulent (velogenic) strains. The IVPI value of the H9 AIV vaccine strain G was 0.50, whereas the IVPI values of the H9 AIV challenge strains were 0.30-0.50, classifying them as low pathogenic AIV strains. The egg infective dose 50 (EID_50_) values of the three NDV isolates were between 8.5 log_10_ EID_50_/0.1 mL and 9.3 log_10_ EID_50_/0.1 mL, and for the H9N2 viruses were between 7.5 log_10_ EID_50_/0.1 mL and 8.0 log_10_ EID_50_/0.1 mL.

### Inactivation confirmation of the viruses

To confirm the complete inactivation of two viruses, the formalin-treated viruses were performed three passages in 10-day-old embryonated SPF chicken eggs. All chicken embryos injected with formalin-treated viruses survived after 120 h, and no HA titer was detected.

### Dynamics of the HI antibody

**Fig. 2:**
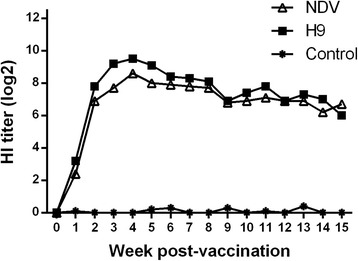
Antibody dynamics of vaccinated chickens. Three-week-old chickens were immunized by subcutaneous injection with the developed inactivated vaccine (NDV, aSG10 and H9N2, G). The mean HI titers for sera collected weekly for 15 weeks post-vaccination are shown

To determine the dynamics of the HI antibody in immunized birds, serum was collected from vaccinated chickens weekly and subjected to a HI test. The antibody titer increased rapidly after inoculation of the inactivated vaccine, and the mean HI titer reached 6.9 log_2_ against NDV and 7.8 log_2_ against H9N2 antigen at 2 wpv. The highest HI titer was observed at 4 wpv, with a mean log_2_ HI titer of 8.6 for NDV and 9.5 for H9N2. Up until 15 wpv, HI titers were still detectable at a high level, with a mean HI titer of 6.7 log_2_ against NDV and 6.0 log_2_ against H9N2 (Fig. 2).

### Protective efficacy against virulent NDV

**Fig. 3:**
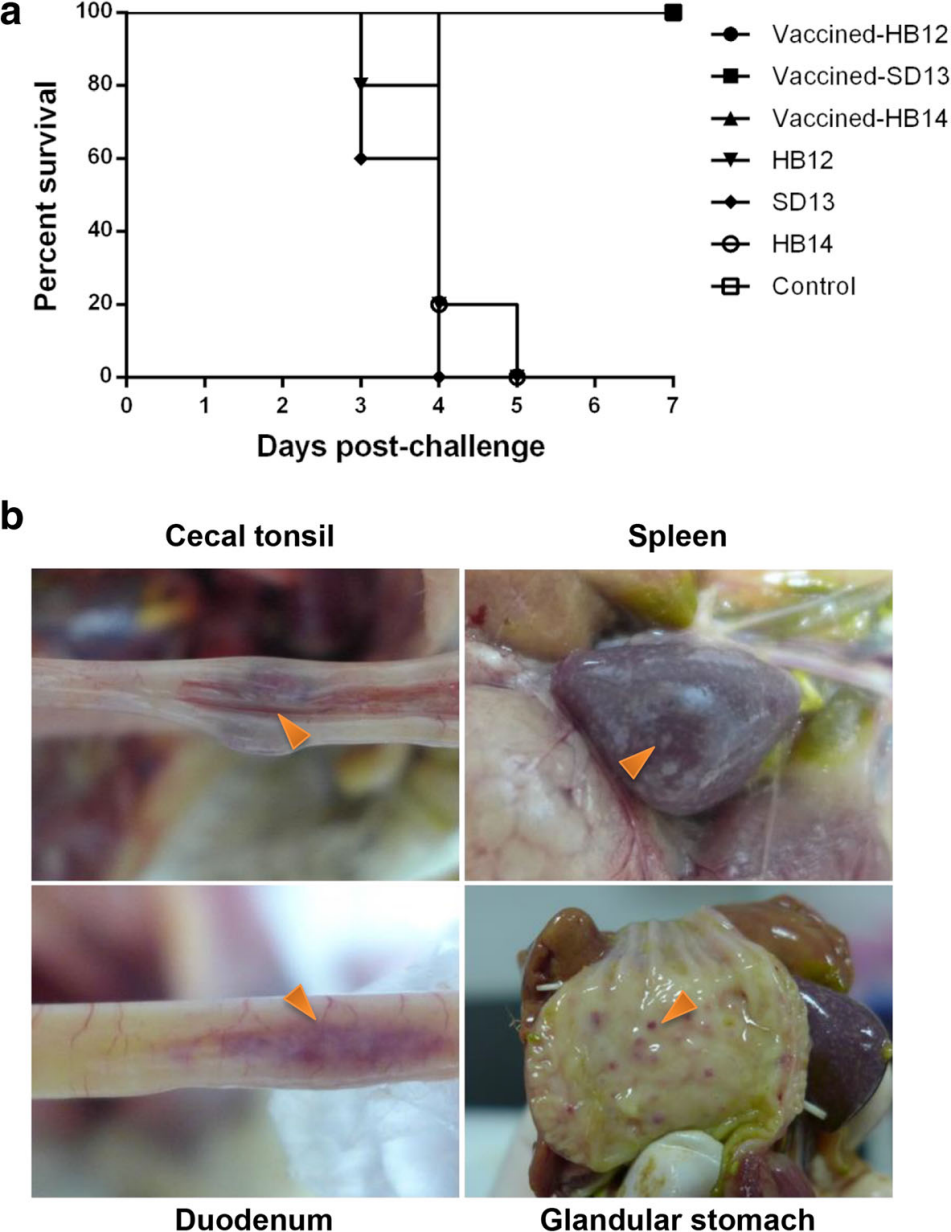
**a** Survival of chickens after challenge with NDV strains isolated in 2012–2014. **b** Gross lesions in the cecal tonsil, spleen, duodenum, and glandular stomach of dead chickens

To evaluate the efficacy of the inactivated vaccine against virulent NDV, three genotype VII NDVs were selected for a challenge test. About 24 h post-challenge, all of the control birds showed the typical clinical signs of infection, including depression, ruffled feathers, greenish diarrhea, and trembling, and within 5 days post-challenge a 100% mortality rate was observed (Fig. 3A). Post-mortem examination of the dead birds revealed characteristic lesions of ND such as petechiae in the proventriculus, white punctate necrosis in the spleen, and hemorrhages in the intestine and cecal tonsil (Fig. 3b). By contrast, there were no clinical signs, gross lesions, or mortality even after 14 days post-challenge in vaccinated chickens. At day 5, virus shedding was detected from all of the chickens that died in all of the unvaccinated groups (3.01–3.64 log_10_ EID_50_ /0.1 mL), no NDV shedding was recorded in group B, and virus shedding was only detected in one of 10 birds in vaccinated groups A (log_10_ EID_50_ /0.1 mL < 1.00) and C (log_10_ EID_50_ /0.1 mL = 1.07), as shown in Table 2.

**Table 2:** Immune protection against NDV isolated in 2012–2014

Virus for challenge	Group	HI titer (log2)	Clinical signs/total	Deaths/total	Positive swabs/total	Positive swabs viral loading (EID_50_/0.1 mL)	Protection rate (%)^a^
NDV-HB12	A	7.7	0/10	0/10	1/10^b^	<10^-1.00^	90
	D	0.0	5/5	5/5	5/5	10^-3.01^	0
NDV-SD13	B	7.3	0/10	0/10	0/10	ND	100
	E	0.0	5/5	5/5	5/5	10^-3.52^	0
NDV-HB14	C	7.5	0/10	0/10	1/10	10^-1.07^	90
	F	0.0	5/5	5/5	5/5	10^-3.64^	0
-	M	0.0	0/5	0/5	0/5^b,c^	ND	100

*ND* no data

^a^Protection definition: means survival without signs of clinical disease not shedding

^b^Cloaca swab

^c^Throat swab

### Protective efficacy against LPAI H9N2

To evaluate the efficacy of the inactivated vaccine against prevalent H9N2 viruses, three selected viruses were used for the challenge test. When challenged with H9N2, no specific clinical signs were observed, with the exception of one bird from each of the control groups J and K, which exhibited signs of depression and death occurred at 6 days post-inoculation. Virus was detected in all of the chickens in the control groups at day 5. No virus shedding was recorded in any of the vaccinated groups (Table 3).

**Table 3:** Immune protection against H9 AIV isolated in 2012–2014

Virus for challenge	Group	HI titer (log2)	Clinical signs/total	Deaths/total	Positive swabs/total	Protection rate (%)^a^
H9-JS12	G	9.2	0/10	0/10	0/10^c^	100
	J	0.0	1/5	1/5	5/5	0
H9-HB13	H	9.3	0/10	0/10	0/10	100
	K	0.0	1/5	1/5	5/5	0
H9-SD14	I	8.7	0/10	0/10	0/10	100
	L	0.0	0/5	0/5	5/5	0
-	M	0.0	0/5	0/5	0/5^b,c^	100

^a^Protection definition: means survival without signs of clinical disease not shedding

^b^Cloaca swab

^c^Throat swab

## Discussion

In China, virulent NDV genotype VII first emerged in the 2000s [23, 24], and strains recently isolated from chickens have also been shown to belong to this genotype. Recent studies and our present results show that isolates from this prevalent genotype differ from the widely used vaccine strain LaSota and B1 in the immune responses they evoke [8, 24]. Therefore, antigenic differences between the prevalent circulating genotype and vaccine strains might account for the current ND outbreaks in vaccinated poultry flocks [25]. To tackle this problem, new vaccines that better match circulating viruses have been developed [23, 26]. The current study therefore aimed to develop an NDV vaccine strain that is immunogenic and safe for use in chickens.

Vaccination is an effective way to prevent and control the spread of H9N2 AIVs, but the current vaccine used in China is prepared from isolates that were circulating in the early 1990s, and their protective efficacy has been decreasing in recent years. A HI test showed that the commercial vaccine strain SS/94 does not provide robust protection against emerging H9N2 AIVs in China [22]. It is therefore crucial that novel candidate vaccine strains are employed to combat emerging H9N2 AIVs in China. Previous phylogenetic analysis of the HA genes of H9N2 AIVs isolated in China between 2010 and 2014 demonstrated that group III, as an emerging lineage, has become one of the dominant clades. Increasing evidence suggests that the antigenic drift of field H9N2 AIV in China is a result of the incomplete protection offered by the commercial vaccine, probably caused by the persistent prevalence of H9N2 AIV. Our results also highlight that the currently used vaccine strain should be regularly evaluated and updated to achieve optimal protection against AIV infections [21].

According to previous phylogenetic analyses, most NDVs isolated in 2010–2014 belonged to genotype VII and most H9N2 AIVs belonged to subgroup III in the BJ/94-like clade, a predominant genotype. These strains showed high homology to each other and a lower level of homology to traditional vaccine strains in China. In this study, a new inactivated vaccine was developed based on the prevalent NDV and H9N2 AIV strains (NDV aSG10 and H9N2 G) and its efficacy was evaluated in chickens [27, 28].

Evaluation of NDV or AIV vaccine immunogenicity is largely based on HI antibody titers. In this study, the HI antibody titers of all chickens vaccinated with the inactivated vaccine were detected at weekly intervals until 15 wpv by the HI test. The results indicated that the vaccine could induce a fast immunological reaction and the mean HI titers were over 6.9 log_2_ against both NDV and H9N2 AIV at 2 wpv. At 4 wpv, peak levels of HI antibodies were detected with a mean HI titer of 8.6 log_2_ against NDV and 9.5 log_2_ against H9N2. Antibody titers of more than 6.0 log_2_ were detected up until at least 15 wpv. The results suggested that this new inactivated vaccine was significantly more effective at generating HI antibody responses than the traditional vaccine strain.

We next evaluated whether vaccination of chickens with the inactivated NDV and H9N2 vaccine could reduce the disease rates of both endemic NDV and H9N2 viruses in China by challenge test. We challenged the birds via the injection route in order to the birds receive actual dose of challenge viruses. We also tried a natural route for NDV and H9N2 in our previous studies and the birds demanded higher HI antibodies to obtain good protection [28, 29]. Reduced mortality rates and decreased viral shedding were detected. Unvaccinated chickens challenged with NDV showed a 100% mortality rate and high levels of virus shedding of 3.01–3.64 log_10_ EID_50_/0.1 mL, compared with no mortality and a lower rate of viral shedding of 0–1.07 log_10_ EID50/0.1 mL in vaccinated groups. All of the unvaccinated chickens challenged with H9N2 shed virus, whereas no virus shedding was detected in vaccinated groups.

## Conclusion

Our results showed that the inactivated NDV and H9N2 vaccine (strain NDV aSG10 and H9N2 G) induces a high and prolonged immune response in vaccinated chickens and good protection efficacy against prevalent viruses. This novel vaccine could therefore potentially be used in the poultry industry to prevent and control ND and H9N2 influenza in chickens in China.

## Methods

### Animals

All SPF chicken eggs and chickens were purchased from Merial Vital Laboratory Animal Technology Co., Ltd (Beijing, China). The chickens were kept in isolators at China Agricultural University throughout the experiment and the animal rearing facilities were approved by the Beijing Administration Committee of Laboratory Animals under the leadership of the Beijing Association for Science and Technology (approval ID SYXK [Jing] 2013–0013).

### Vaccine viruses

The selected NDV candidate was strain Chicken/China/Shandong/aSG10/2010 (abbreviated as aSG10) of genotype VIId, which was an attenuated form of the virulent strain SG10 isolated from an outbreak of ND in chickens with an intracerebral pathogenicity index (ICPI) of 1.89 and a mean death time of 45 h [29]. The selected H9N2 AIV candidate was strain A/chicken/Hebei/G/2012 (abbreviated as G) of BJ/94 (subgroup III), which was isolated from a vaccinated breeder broiler flock in 2012 in northern of China.

### Challenge viruses

HA gene segments of 19 H9N2 viruses and F gene segments of 11 NDVs isolated in China from 2012 to 2014 were sequenced as described previously [21, 27]. Multiple sequence alignment was conducted using the Clustal W program of the MEGA 5.1 software. A phylogenetic tree was constructed using the neighbor-joining method with 1000 bootstrap replicates using the MEGA 5.1 software. According to phylogenetic analyses, three NDV and H9N2 virus isolates were chosen for challenge experiments.

### Biological characterization of the viruses

Each virus was amplified in 10-day-old SPF embryonated chicken eggs, and the virus titer (EID_50_) was determined and calculated by the Reed–Muench method [30] based on a hemagglutination assay of the allantoic fluid of eggs inoculated with 10-fold serial dilutions of viruses. The virulence of the viruses was evaluated using standard pathogenicity tests, the ICPI [31] for NDVs, and the IVPI [6] for H9N2 AIVs.

### Vaccine development

NDV strains aSG10 and H9N2 G were propagated in the allantoic cavities of 10-day-old SPF embryonated chicken eggs. Allantoic fluid was harvested 72–96 h after inoculation, and the virus titer in the allantoic fluid of 10^8.50^ EID_50_/0.1 mL for two virus was used for antigen production. The egg-derived virus was inactivated with formalin at 37 °C at a final concentration of 0.1% for 18 h for NDV aSG10 or 0.2% for 24 h for H9N2 G. Complete inactivation of the virus was confirmed through three passages in 10-day-old embryonated SPF chicken eggs. To produce the vaccine, the inactivated viral antigens were blended at a ratio of 1:1 (V/V), then mixed with oil emulsion adjuvants Marcol 52 (Seppic, Paris, France) at a ratio of 1:2 (V/V).

### Efficacy experiment

#### Dynamics of HI antibody

Forty 3-week-old SPF While Leghorn chickens were divided into two groups and 20 for each. The birds in Group 1 were vaccinated with one single dose (0.25 mL/bird) of the inactivated vaccine via subcutaneous injection. Group 2 was the control. Blood was collected from each group (n = 10) at weekly intervals until 15 weeks post-vaccination (wpv). Serum was separated and stored at −20 °C until use. The antibody titers were determined by HI tests using 1% chicken red blood cells according to the OIE recommended protocol [32]. The HI titers were expressed as the log2 of the reciprocal of the highest serum dilution, resulting in complete inhibition of red blood cells.

#### Challenge of vaccinated chickens with LPAI H9N2 and virulent NDV

SPF chickens were challenged with one of three LPAI H9N2 avian influenza viruses or one of three virulent NDV challenge strains three weeks after vaccination and observed for clinical disease and shedding for two weeks after challenge. A total of 95, 3-week-old, SPF chickens were randomly divided into 13 groups (A–M) and treated as shown in Table 4. In brief, six groups (A–C and G–I, n = 10) were injected subcutaneously with one single dose (0.25 mL/bird) of the inactivated vaccine, another seven unimmunized groups served as challenged controls (D–F and J–L, n = 5) and unchallenged controls (M, n = 5). Chickens of six immunized groups and six control groups were infected with 10^5.0^ EID_50_ for NDV (Intramuscular injection) and 10^6.0^ EID_50_ for H9N2 (Intravenous injection) at 3 wpv. Chickens were monitored daily for morbidity and mortality for 14 days.

**Table 4:** Assignment of group and the treatment of chickens

Group	No. of birds	Vaccination^a^	Challenge	Dose	Route
A	*n* = 10	Yes	NDV-HB12	10^5.0^EID_50_	Intramuscular injection
B	*n* = 10	Yes	NDV-SD13		
C	*n* = 10	Yes	NDV-HB14		
D	*n* = 5	No	NDV-HB12		
E	*n* = 5	No	NDV-SD13		
F	*n* = 5	No	NDV-HB14		
G	*n* = 10	Yes	H9-JS12	10^6.0^EID_50_	Intravenous injection
H	*n* = 10	Yes	H9-HB13		
I	*n* = 10	Yes	H9-SD14		
J	*n* = 5	No	H9-JS12		
K	*n* = 5	No	H9-HB13		
L	*n* = 5	No	H9-SD14		
M	*n* = 5	No	No	No	No

^a^Subcutaneous injection

#### Virus isolation and titration

To determine viral shedding from individual chickens, cloacal or throat swabs were obtained at 5 days post-challenge for virus isolation in eggs. Swab samples were suspended in 1.0 mL of PBS supplemented with penicillin-streptomycin (10,000 IU/mL) and then inoculated into 10-day-old embryonated chicken eggs (n = 3 eggs/sample) through the intra-allantoic route. The allantoic fluid was harvested at 5 days post-inoculation and checked for NDV and H9 AIV growth by a HA test using 1% chicken red blood cells. Positive samples were further titrated by EID_50_ determination in 10-day-old embryonated chicken eggs.

## Abbreviations

AIV: Avian influenza virus
EID_50_: Egg infective dose 50
F: Fusion
HA: Hemagglutinin
HI: Hemagglutination inhibition
ICPI: Intracerebral pathogenicity index
IVPI: Intravenous pathogenicity index
ND: Newcastle disease
NDV: Newcastle disease virus
wpv: Weeks post-vaccination
